# Dynamic Alterations in the Gut Microbiota of Collagen-Induced Arthritis Rats Following the Prolonged Administration of Total Glucosides of Paeony

**DOI:** 10.3389/fcimb.2019.00204

**Published:** 2019-06-12

**Authors:** Jine Peng, Xuran Lu, Kaili Xie, Yongsong Xu, Rui He, Li Guo, Yaxin Han, Sha Wu, Xuerong Dong, Yun Lu, Zhengyue Liu, Wei Cao, Muxin Gong

**Affiliations:** ^1^School of Traditional Chinese Medicine, Capital Medical University, Beijing, China; ^2^Beijing Key Laboratory of Traditional Chinese Medicine Collateral Disease Theory Research, Beijing, China; ^3^Department of Rheumatology, Guang'anmen Hospital, China Academy of Chinese Medical Sciences, Beijing, China

**Keywords:** collagen-induced arthritis, gut microbiota, immunity, rheumatoid arthritis, total glucosides of paeony

## Abstract

Rheumatoid arthritis (RA) is a common autoimmune disease linked to chronic inflammation. Dysbiosis of the gut microbiota has been proposed to contribute to the risk of RA, and a large number of researchers have investigated the gut-joint axis hypothesis using the collagen-induced arthritis (CIA) rats. However, previous studies mainly involved short-term experiments; very few used the CIA model to investigate changes in gut microbiota over time. Moreover, previous research failed to use the CIA model to carry out detailed investigations of the effects of drug treatments upon inflammation in the joints, hyperplasia of the synovium, imbalance in the ratios of Th1/Th2 and Th17/Treg cells, intestinal cytokines and the gut microbiota following long-term intervention. In the present study, we carried out a 16-week experiment to investigate changes in the gut microbiota of CIA rats, and evaluated the modulatory effect of total glucosides of paeony (TGP), an immunomodulatory agent widely used in the treatment of RA, after 12 weeks of administration. We found that taxonomic differences developed in the microbial structure between the CIA group and the Control group. Furthermore, the administration of TGP was able to correct 78% of these taxonomic differences, while also increase the relative abundance of certain forms of beneficial symbiotic bacteria. By the end of the experiment, TGP had reduced body weight, thymus index and inflammatory cell infiltration in the ankle joint of CIA rats. Furthermore, the administration of TGP had down-regulated the synovial content of VEGF and the levels of Th1 cells and Th17 cells in CIA rats, and up-regulated the levels of Th2 cells and Treg cells. The administration of TGP also inhibited the levels of intestinal cytokines, secretory immunoglobulin A (SIgA) and Interferon-γ (IFN-γ). In conclusion, the influence of TGP on dynamic changes in gut microbiota, along with the observed improvement of indicators related to CIA symptoms during 12 weeks of administration, supported the hypothesis that the microbiome may play a role in TGP-mediated therapeutic effects in CIA rats. The present study also indicated that the mechanism underlying these effects may be related to the regulation of intestinal mucosal immunity remains unknown and deserves further research attention.

## Introduction

Rheumatoid arthritis (RA) is a chronic and inflammatory autoimmune disease which is characterized by synovial tissue hyperplasia, vasospasm and structural damage to the ligaments, bones and cartilage. Approximately 0.3–1% of the global population is affected by RA (Chaudhari et al., 2016); the age-adjusted prevalence of RA in China is reported to be 0.28% (Li et al., 2012).

Over recent years, a growing body of evidence has developed in support of a potential relationship between the gut microbiota and RA. For example, Scher et al. (2013) identified a strong association between *Prevotella copri* and new-onset untreated RA (NORA). Chen et al. (2016) further noted that patients with RA had a reduced diversity of gut microbiota and that this finding correlated with the duration of disease and the levels of auto-antibodies. Other reports have highlighted that improvements in RA are related to the regulation of gut microbiota, the composition of the gut microbiota may serve as a biomarker for treatment success, and has been correlated with improvements in the overall symptoms of CIA mice (Ben-Amram et al., 2017; Xiao et al., 2018). For example, the susceptibility and severity of arthritis in a variety of rodent strains was shown to be reduced when animals were maintained in germ-free environments, or in environments with restricted bacterial flora (Liu et al., 2016). Furthermore, supplementation with *Lactobacillus casei 01* was shown to improve disease activity and inflammatory status in patients with RA (Vaghef-Mehrabany et al., 2014). Zhang et al. (2015) also reported concordant dysbiosis of both fecal and oral samples from RA patients, which was partially resolved following RA treatment.

Total glucosides of paeony (TGP) are widely used for the treatment of RA in China and are known to act as an immunomodulatory agent. TGP is routinely extracted from the roots of *Paeonia lactiflora* Pall, a Chinese traditional herbal medicine (CTM). The main chemical constituents of TGP are paeoniflorin, albiflorin, hydroxy-paeoniflorin, paeonin, and benzoylpaeoniflorin; these belong to the family of monoterpene glycosides. These components exhibit low levels of bioavailability (Takeda et al., 1995, 1997; Fei et al., 2016); this is because they show poor levels of absorption (Chen et al., 1999; Xia et al., 2007), can readily accumulate in the gastrointestinal tract (Zhang et al., 2012; Zhao and Wang, 2014; Sun et al., 2017) and can be transformed by the gut microbiota (Takeda et al., 1997; Tong et al., 2010). Conversely, TGP may also influence the gut microbiota. However, it remains unknown as to whether gut microbiota play a role in the TGP-mediated treatment of CIA rats. In the present study, the high-throughput 16S rRNA gene sequencing technology was used to investigate the effect of TGP upon temporal changes in the gut microbiota of CIA rats over a prolonged period of administration. This experiment was carried out to prove whether the gut microbiota plays a role in the TGP-mediated amelioration of CIA symptoms and to identify the key genera involved.

Although the pathophysiology of RA is not yet completely understood, vascular endothelial growth factor (VEGF) has been identified as the critical angiogenic factor responsible for vascular proliferation in RA and the invasion of blood vessels into the synovial lining membrane (Lee and Bae, 2018). Other reports hypothesize that the imbalance of Th1/Th2 cells and Th17/Treg cells in the peripheral blood mononuclear cells represent key factors in the development/prevention of RA (Lina et al., 2011). However, general immunity may not provide the sole primary trigger for RA; it is also possible that localized autoimmune processes upon the intestinal mucosal surfaces may also act as potential triggers (Mankia and Emery, 2015). As the largest mucosal surface in mammals, the intestinal tract represents the major communication link with the external environment and is constantly exposed to potential pathogens and beneficial commensal microorganisms (Artis, 2008). SIgA is known to play an important role in the immune defense function of the intestinal mucosa, and causes microorganisms to agglutinate, thus preventing their attachment to host epithelial cells and limiting the possibility for bacterial colonization (Williams and Gibbons, 1972; Wold et al., 1990; Wershil and Furuta, 2008). IFN-γ is an important factor in promoting B cell differentiation and the secretion of SIgA (Kjerrulf et al., 1997). Previous researchers found that levels of SIgA within the intestinal contents of CIA rats were increased significantly, as were the levels of IFN-γ in the intestinal tissue (Wang et al., 2015). Previous studies of TGP in the treatment of RA, which were carried out over short term periods, showed that TGP can inhibit the production of VEGF and ameliorate the abnormal proliferation of synoviocytes in CIA rats (Zhu et al., 2005; Deng et al., 2010; Zhang and Dai, 2012); TGP was also shown to reduce the differentiation of Th1 and Th17 cells in CIA mice (Lin et al., 2012). However, RA patients usually take TGP (1.2 ~ 1.8 g·d^−1^) orally for 8 weeks during a typical course of treatment. This treatment results in a slight curative effect after 2 weeks; obvious therapeutic effect only appears by week 8 of administration. After taking TGP for 16 to 24 weeks, the effect increased slightly but then stabilized, although it could still prolong the duration of efficacy following drug withdrawal (Wang et al., 1994). In the present study, a combination of histopathology, flow cytometry and enzyme-linked immunosorbent assay (ELISA) were utilized to investigate changes in the ankle, synovium, peripheral immune function and intestinal factors in CIA rats following the administration of TGP for 12 weeks.

## Materials and Methods

### Animals

Seven- to eight-week male Sprague-Dawley (SD) rats (weighing 160–200 g) were obtained from the Beijing Vital River Laboratory Animal Technology Co. Ltd. (Beijing, China, License no. SCXK 2016-0011).

### Induction of CIA and Animal Grouping

Thirty-six untreated SD rats were randomly divided into 4 groups (*n* = 9): a normal control group (Control), a low-dose control TGP group (C+LTGP), a medium-dose control TGP group (C+MTGP) and a high-dose control TGP group (C+HTGP). TGP was purchased from Ningbo Liwah Pharmaceutical Co. Ltd. (National Pharmaceutical Standard H20055058, batch number 161118). Sixty SD rats were induced with a 100 μL subcutaneous injection of 1 mg/mL bovine type II collagen (Chondrex, Catalog #20022, Lot: 60425, USA) emulsified with complete Freund's adjuvant (Chondrex, Catalog #7001, Lot: 60410, USA). On day 21, rats also received a 100 μL subcutaneous booster dose of 1 mg/mL bovine type II collagen emulsified with incomplete Freund's adjuvant (Chondrex, Catalog #7002, Lot: 160417, USA) (Trentham et al., 1977; Brand et al., 2007; Jia et al., 2016).

After the induction of inflammation, the degree of joint disease was observed and recorded. According to a 5-point scale, the Arthritis index (AI) was calculated as follows: 0 = no evidence of erythema and swelling; 1 = mild erythema and swelling of the wrist or ankle; 2 = moderate erythema and swelling from the wrist to the metacarpal joints or from the ankle to the metatarsal joints; 3 = severe erythema and swelling of the entire paw including the digits and 4 = maximal erythema and swelling of the paw, or ankylosis of the limb. The total AI was the sum of the scores for each of the four limbs (Marcinska et al., 2016).

**Figure 1:**
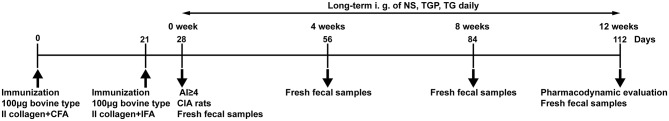
Experimental schedule. Male SD rats were immunized by subcutaneous injection of with 100 μg bovine type II collagen emulsified with CFA on day 0. On day 21, rats received a subcutaneous booster 100 μg bovine type II collagen emulsified with IFA. On Day 28, the rats with AI ≥ 4 in the model rats were regarded as CIA rats. Fresh fecal samples of the rats were collected at day 28, day 56, day 84, and day 112. Long-term intragastric administration of NS, TGP, TG daily, and do pharmacodynamic evaluation at the end point of experiment.

On Day 28, rats with an AI ≥ 4 were regarded as having CIA. According to the AI scores, and body weight, the CIA rats were randomly divided into 5 groups (*n* = 10): a CIA control group (CIA), a low-dose CIA TGP group (CIA+LTGP), a medium-dose CIA TGP group (CIA+MTGP), a high-dose CIA TGP group (CIA+HTGP) and a CIA+TG (tripterygium glycosides) group. TG was purchased from Hunan Qianjin Xieli Pharmaceutical Co. Ltd. (National Pharmaceutical Standard Z43020138, batch number 20161002). We ensured that there were no significant differences in body weight or AI scores between these 5 groups of CIA rats. The low, medium and high doses of TGP were 158 mg/kg (a clinical equivalent dose), 474 mg/kg (3 times the clinical equivalent dose), and 948 mg/kg (6 times the clinical equivalent dose), respectively. The dose of TG was 7.89 mg/kg (a clinical equivalent dose) and the drugs were administered continuously for 12 weeks. The experimental schedules are shown in Figure 1.

### Microbiota DNA Sequencing

Fresh fecal samples of the rats were collected at 0, 4, 8, 12 weeks as shown in Figure 1, then stored at −80°C. Total microbial genomic DNA samples were extracted using the Mag-Bind soil DNA kit (200) (M5635-02, OMEGA, USA), following the manufacturer's instructions, and stored at −20°C prior to further analysis. The quantity and quality of extracted DNAs were measured using a NanoDrop ND-1000 spectrophotometer (Thermo Fisher Scientific, Waltham, MA, USA) and agarose gel electrophoresis, respectively. PCR amplification of the bacterial 16S rRNA genes V3–V4 region was performed using the forward primer 338F (5′-ACTCCTACGGGAGGCAGCA-3′) and reverse primer 806R (5′-GGACTACHVGGGTWTCTAAT-3′). Sample-specific 7-bp barcodes were incorporated into the primers for multiplex sequencing. The PCR components contained 5 μL of Q5 reaction buffer (5×), 5 μL of Q5 High-Fidelity GC buffer (5×), 0.25 μL of Q5 High-Fidelity DNA Polymerase (5 U/μL), 2 μL (2.5 mM) of dNTPs, 1 μL (10 μM) of each Forward and Reverse primer, 2 μL of DNA Template, and 8.75 μL of ddH_2_O.Thermal cycling consisted of initial denaturation at 98°C for 5 min, followed by 24 cycles consisting of denaturation at 98°C for 30 s, annealing at 50°C for 30 s, and extension at 72°C for 60 s, with a final extension of 10 min at 72°C. PCR amplicons were purified with Agencourt AMPure Beads (Beckman Coulter, Indianapolis, IN) and quantified using the PicoGreen dsDNA Assay Kit (Invitrogen, Carlsbad, CA, USA). After the individual quantification step, amplicons were pooled in equal amounts, and paired-end 2 ×300 bp sequencing was performed using the Illlumina MiSeq platform with MiSeq Reagent Kit v3.

### Bioinformatic Analysis

Quantitative Insights Into Microbial Ecology (QIIME, version 1.8) software was used to quality filter raw sequences (Caporaso et al., 2010). Paired-end reads were assembled using FLASH (Magoč and Salzberg, 2011). Following the detection of chimeras, the remaining high-quality sequences were clustered into operational taxonomic units (OTUs) at 97% sequence identity by UCLUST (Edgar, 2010). A representative sequence was selected from each OTU using default parameters. OTU taxonomic classification was then conducted by BLAST searching the representative sequences set against the Green Genes Database (DeSantis et al., 2006) using the best hit (Altschul et al., 1997).

Sequence data analyses were mainly performed using QIIME and R packages (version 3.2). OTU-level alpha diversity indices, such as Chao1 richness estimator and Shannon diversity index, were calculated using the OTU table produced by QIIME. OTU-level ranked abundance curves were then generated to compare the richness and evenness of OTUs among samples. A Venn diagram was generated to visualize the shared and unique OTUs among samples or groups using the R package and based upon the occurrence of OTUs across samples/groups regardless of their relative abundance (Zaura et al., 2009). The significance of differentiation in the microbiota structure among groups was assessed by analysis of similarities (ANOSIM) (Clarke, 1993; Warton et al., 2012) using the “vegan” function in the R package. Partial least squares discriminant analysis (PLS-DA) was also introduced as a supervised model with which to reveal the variation in microbiota among groups, using the “plsda” function in the R package “mixOmics” (Chen et al., 2011). Linear discriminant analysis effect size (LEfSe) was then performed to detect differentially abundant taxa across groups using default parameters (Segata et al., 2011). The abundance of different taxa within samples at the phylum, class, order, family and genus levels were investigated statistically using taxonomic composition analysis (http://qiime.org/). Microbial functions were predicted by the phylogenetic investigation of communities by reconstructing unobserved states (PICRUSt) based upon high-quality sequences (Langille et al., 2013).

### Body Weight and Organ Index

Rats were given a TGP by gavage daily for 12 weeks, and the body weight of the rats in each group were monitored dynamically. At the end point of the experiments, the weights of fresh and clean thymus and spleen, as well as body weight were recorded. Organ indices for the thymus and spleen were then calculated using the following formula.

$$\begin{array}{r} Organ\;index\;=\;\frac{\text{organ weight}}{\text{body weight}}\;\times \;100\% \end{array}$$

### Histological Assessment

The hind paws of the animals were removed post-mortem, stored in 10% neutral formalin, decalcified in 20% EDTA for 6 weeks, and then dehydrated and embedded in paraffin. Next, sections were cut along the longitudinal axis, mounted, and stained with hematoxylin and eosin (H&E) to examine the changes of ankle joint. The histological examinations were performed in a blinded fashion for changes in cartilage damage, synovial hypertrophy and inflammatory cell infiltration as previously described (Larsson et al., 2004; Gan et al., 2015), and a composite score summed histological score was calculated. For each paw, these three parameters were each assessed using a 4-point scale (0 = normal; 1 = mild; 2 = moderate; and 3 = maximal). Briefly, for cartilage damage, 0 = normal appearance; 1 = mild destruction of the cartilage surface; 2 = moderate destruction of the cartilage surface; 3 = severe destruction of the cartilage surface. For synovial hypertrophy, 0 = normal appearance, uninflamed appearance of the synovium; 1 = mild localized synovial hypertrophy; 2 = moderate localized synovial hypertrophy; 3 = severe extensive synovial hypertrophy. For inflammatory cell infiltration, 0 = normal appearance; 1 = mild focal infiltration; 2 = moderate focal infiltration; 3 = severe extensive infiltration.

Immunohistochemistry was also performed to detect immunoreactivity for VEGF in the synovium; these assays used a primary VEGF antibody (1:1,000; GB11034, Servicebio, China) and a goat anti-rat IgG as secondary antibody (K5007, DAKO, Denmark). Positive signals were detected with 3, 3′-diaminobenzidine (DAB) (G1211, Servicebio, China). Slides were counterstained with hematoxylin (G1040, Servicebio, China) and positive cells identified by the presence of brown particles. Five random fields were evaluated from each section and Image-Pro Plus 6.0 software (Media Cybernetics Inc., MD, USA) was used to quantify the proportion (in %) of VEGF-positive cells.

### Flow Cytometry

Peripheral blood mononuclear cell suspensions were analyzed using BD LSRFortessa custom flow cytometer (LSRFortessa SORP, Becton, Dickinson and Company), using the following antibodies: Rat CD4 APC-Cy7 OX-35, MS IGG2A KPA ITCL APC-CY7 G155-178, Rat IL-4 PE OX-81, Ms IgG1 Kpa ItCl PE MOPC-21, Rat IFN-Gma FITC DB-1, Ms IgG1 Kpa ItCl FITC MOPC-21, Rat CD25 BV421 OX-39, Mouse IgG1 Kpa ItCl BV421 X40 (BD Pharmingen, USA), Anti-Mouse/Rat IL-17A PerCP-Cyanine5.5, Rat IgG2a K Isotype Control PerCP-Cyanine5.5, Anti-Mouse/Rat Foxp3 APC, Rat IgG2a K Isotype Control APC (eBioscience, USA), and Transcription Factor Buffer Set, Leuko Act Cktl With GolgiPlug (BD Pharmingen, USA).The expression levels of Th1, Th2, Th17, and Treg cells were detected by the expression levels of CD3^+^CD4^+^IFN-γ^+^, CD3^+^CD4^+^IL-4^+^, CD3^+^CD4^+^IL-17A^+^, CD4^+^CD25^+^Foxp3^+^, respectively.

### Evaluation of Intestinal Cytokines

Levels of SIgA were determined in contents recovered from the small intestine, and levels of IFN-γ were determined in tissue samples taken from the small intestine; these tests were carried out with rat SIgA and IFN-γ ELISA kits (Nanjing Jiancheng Bioengineering Research Institute, China). At the end of the experiment, small intestine from each rat were collected and its content were weighed. Normal saline was added to a ratio of 1:9 (mass: volume); this was then vortex-mixed and centrifuged at 3,500 rpm at 4°C for 20 min; the supernatant was then collected for quantitative measurement. For each rat, the entire small intestine was also dissected. This was then rinsed in normal saline. Excess fluid was then removed by blotting the tissue with filter paper. Next, the small intestine was weighed and normal saline added to a ratio of 1:9 (mass: volume). The small intestine was then ground with a homogenizer (PRO200, USA), centrifuged at 3,500 rpm at 4°C for 20 min and the supernatant was collected for quantification. Standard curves for SIgA or IFN-γ concentration were created in accordance with instructions provided with the SIgA and IFN-γ kits. Finally, supernatants were diluted with normal saline to identify the most suitable dilution for measurement by the SIgA or IFN-γ ELISA kit; this ensured that the final detection results fell within the range of the appropriate standard curve.

### Statistical Analysis

Data are shown as mean ± standard error of the mean (SEM). Raw data were first assessed to ensure that the data fitted a normal distribution. If data were normally distributed, and variance was homogenous, then the least significant difference (LSD) method was used to perform analysis of variance (ANOVA) across different experimental groups. Otherwise, the rank sum test was used. Body weight data were analyzed by repeated measures ANOVA. Non-parametric test was used to analyze histological score. All statistical tests were performed using bilateral tests and data were analyzed by SPSS 17 (IBM, Armonk, NY, USA) and Prism 7 (GraphPad, CA, USA) software. *P*-values <0.05 were considered to be statistically significant. Multiple comparisons, based upon the Benjamini & Hochberg method, were deployed in Prism 7 software to control the false discovery rate.

## Results

### Temporal Effects of CIA Upon Gut Microbial Composition and the Correction of Dysbiosis in the Gut Microbiota Following the Administration of TGP in CIA Rats

Analysis of Chao1 and Shannon indices indicated that the sequencing depth covered new phylotypes and most of the diversity (Supplementary Figures 1A–D). Temporal changes in the microbial richness of different experimental groups are depicted in Venn diagrams (Supplementary Figures 2A–D). At 0 and 8 weeks, the number of OTUs in the CIA group were more than that in the Control group, while at 4 and 12 weeks, the number of OTUs in the CIA group was less than that in the Control group. These data suggested that the structure of the gut microbiota may change periodically. Simultaneously, the number of OTUs in TGP-treated CIA groups was more than that in the CIA group after 4, 8, and 12 weeks of TGP intervention, which indicated that TGP could increase the diversity of gut microbiota in CIA rats. To measure the level of similarity between gut microbial communities, ANOSIM of the unweighted UniFrac distance matrix and PLS-DA were performed. At 0, 4, 8, and 12 weeks, ANOSIM and PLS-DA (Supplementary Figures 3A–D) revealed an apparent separation in the structure of the gut microbiota in each group.

**Figure 2:**
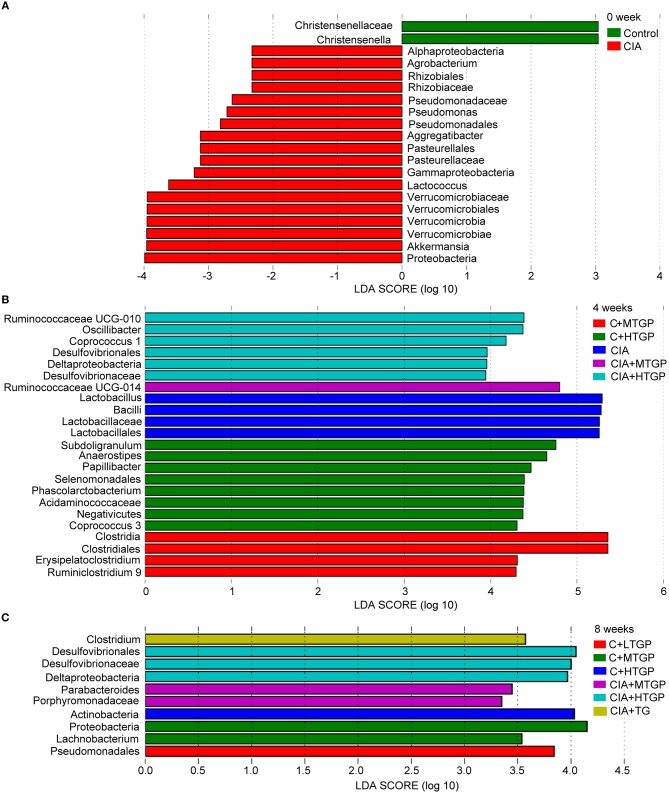
LEfSe revealed the temporal changes of gut microbial community. **(A–C)** LEfSe method was performed to compare taxa among each group at 0, 4, and 8 weeks, respectively. The bar plot lists the significantly differential taxa (*p* < 0.05) based on effect size (LDA score (log 10) > ± 2). Control is the normal control group treated with just normal saline. C+LTGP, C+MTGP and C+HTGP are the control groups treated with 158, 474, and 948 mg/kg TGP, respectively. CIA is the collagen-induced arthritis group treated with just normal saline. CIA+ LTGP, C+MTGP and C+HTGP are the collagen-induced arthritis groups treated with 158, 474, and 948 mg/kg TGP, respectively. CIA+TG is the collagen-induced arthritis group treated with 7.89 mg/kg TG.

To identify temporal changes in the fecal microbiota community, LEfSe was used to compare the microbial species present in the guts of rats harvested from each group (Figure 2). A key genus, *Christensenella*, belonging to the Christensenellaceae, was most abundant in the Control group. However, there were six genera that showed the highest relative abundance in the CIA group: *Akkermansia* originating from Verrucomicrobia; *Lactococcus* and *Lactobacillus*, originating from Firmicutes, as well as *Pseudomonas, Aggregatibacter*, and *Agrobacterium*, which originate from Proteobacteria (Figures 2A,B). Following TGP intervention, there were five key genera, and a key family, in the CIA groups of rats treated with TGP. Specifically, *Ruminococcaceae UCG-014* and *Parabacteroides* were most abundant in the CIA+MTGP group, while *Coprococcus 1, Oscillibacter, Ruminococcaceae UCG-010*, and Desulfovibrionaceae were most abundant in the CIA+HTGP group. *Clostridium* was most abundant in the CIA+TG group (Figures 2B,C).

**Figure 3:**
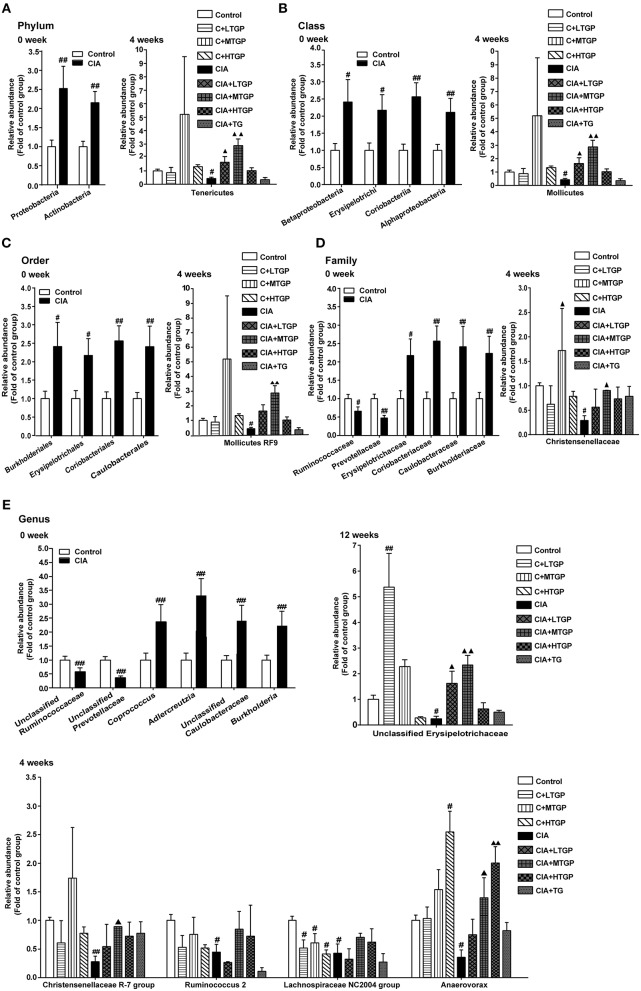
The temporal changes of gut microbial composition at the phylum, class, order, family and genus level in taxonomic composition analysis. **(A)** The relative abundance of the bacterial phylum detected in fecal samples at 0, 4 weeks, respectively. **(B)** The relative abundance of the bacterial class detected in fecal samples at 0, 4 week, respectively. **(C)** The relative abundance of the bacterial order detected in fecal samples at 0, 4 weeks, respectively. **(D)** The relative abundance of the bacterial family detected in fecal samples at 0, 4 weeks, respectively. **(E)** The relative abundance of the bacterial genus detected in fecal samples at 0, 4, 12 weeks, respectively. Control is the normal control group treated with just normal saline. C+LTGP, C+MTGP and C+HTGP are the control groups treated with 158, 474, and 948 mg/kg TGP, respectively. CIA is the collagen-induced arthritis group treated with just normal saline. CIA+ LTGP, C+MTGP and C+HTGP are the collagen-induced arthritis groups treated with 158, 474, and 948 mg/kg TGP, respectively. CIA+TG is the collagen-induced arthritis group treated with 7.89 mg/kg TG. Data indicate mean ± SEM. ^##^*P* <0.01, ^#^*P* <0.05 vs. Control; ^▴▴^*P* <0.01, ^▴^*P* <0.05 vs. CIA, indicating significant differences.

To further profile specific changes occurring in the gut microbiota, we analyzed the relative abundance of the predominant taxa in each group, as identified by sequencing. Figure 3 shows a detailed overview of the gut microbial composition of each group at the phylum, class, order, family and genus level. At the phylum level, compared to the Control group, the CIA group showed a considerably higher abundance of Proteobacteria (*P* <0.01) and Actinobacteria (*P* <0.01), but a reduced abundance of Tenericutes (*p* < 0.05) (Figure 3A). At the class level, the CIA group showed a higher abundance of Betaproteobacteria (*p* < 0.05), Erysipelotrichi (*p* < 0.05), Coriobacteriia (*P* <0.01), and Alphaproteobacteria (*P* <0.01), but a lower abundance of Mollicutes (*p* < 0.05) (Figure 3B). At the order level, Burkholderiales (*p* < 0.05), Erysipelotrichales (*p* < 0.05), Coriobacteriales (*P* <0.01), and Caulobacterales (*P* <0.01) were more abundant in the CIA group than in the Control group. However, Mollicutes RF9 (*p* < 0.05) was less abundant in the CIA group (Figure 3C). At the family level, the CIA group showed an increased abundance of Erysipelotrichaceae (*p* < 0.05), Coriobacteriaceae (*P* <0.01), Caulobacteraceae (*P* <0.01), and Burkholderiaceae (*P* <0.01), but a reduced abundance of Ruminococcaceae (*p* < 0.05), Prevotellaceae (*P* <0.01), and Christensenellaceae (*p* < 0.05) compared to the control group (Figure 3D). At the genus level, there was a higher abundance of Coprococcus (*P* <0.01), Adlercreutzia (*P* <0.01), Unclassified_Caulobacteraceae (*P* <0.01), and Burkholderia (*P* <0.01) in the CIA group. However, Unclassified_Ruminococcaceae (*P* <0.01), Unclassified_Prevotellaceae (*P* <0.01), Christensenellaceae R-7 group (*P* <0.01), Ruminococcus 2 (*p* < 0.05), Lachnospiraceae NC2004 group (*p* < 0.05), Anaerovorax (*p* < 0.05), and Unclassified_Erysipelotrichaceae (*p* < 0.05) were less abundant in the CIA group than in the Control group (Figure 3E). Collectively, these results showed that CIA played an important role in the alteration of gut microbiota. Interestingly, at 8 weeks, there was no significant difference between the CIA group and the Control group at the phylum, class, order, family and genus level. At 12 weeks, there was no significant difference between the CIA group and the Control group at the phylum, class, order and family level. In contrast, after 4, 8, and 12 weeks of TGP intervention, it was apparent that dysbiosis in the gut microbiota had been corrected at least in part. At the phylum level, compared to the CIA group, the relative abundance of Tenericutes had increased in the CIA+LTGP (*p* < 0.05) and the CIA+MTGP (*P* <0.01) groups. At the class level, compared to the CIA group, there was a higher abundance of Mollicutes in the CIA+LTGP (*p* < 0.05) and CIA+MTGP (*P* <0.01) groups. At the order level, compared to the CIA group, the relative abundance of Mollicutes RF9 had increased in the CIA+MTGP (*P* <0.01) group. At the family level, Christensenellaceae (*p* < 0.05) was significantly more abundant in the CIA+MTGP group than in the CIA group. At the genus level, the relative abundance of Unclassified_Erysipelotrichaceae (*p* < 0.05) had increased in the CIA+LTGP group and the relative abundance of Christensenellaceae R-7 (*p* < 0.05), Anaerovorax (*p* < 0.05) and Unclassified_Erysipelotrichaceae (*P* <0.01) had increased in the CIA+MTGP group. The relative abundance of Anaerovorax had also increased in the CIA+HTGP group (*P* <0.01). Meanwhile, at the genus level, compared to the Control group, the abundance of Lachnospiraceae NC2004 had decreased in the C+LTGP (*p* < 0.05), C+MTGP(*p* < 0.05) and C+HTGP (*p* < 0.05) groups, while the abundance of Unclassified_Erysipelotrichaceae had increased in the C+LTGP group (*p* < 0.05). The abundance of Anaerovorax had significantly increased in the C+HTGP group (*p* < 0.05) (Figures 3A–E).

**Figure 4:**
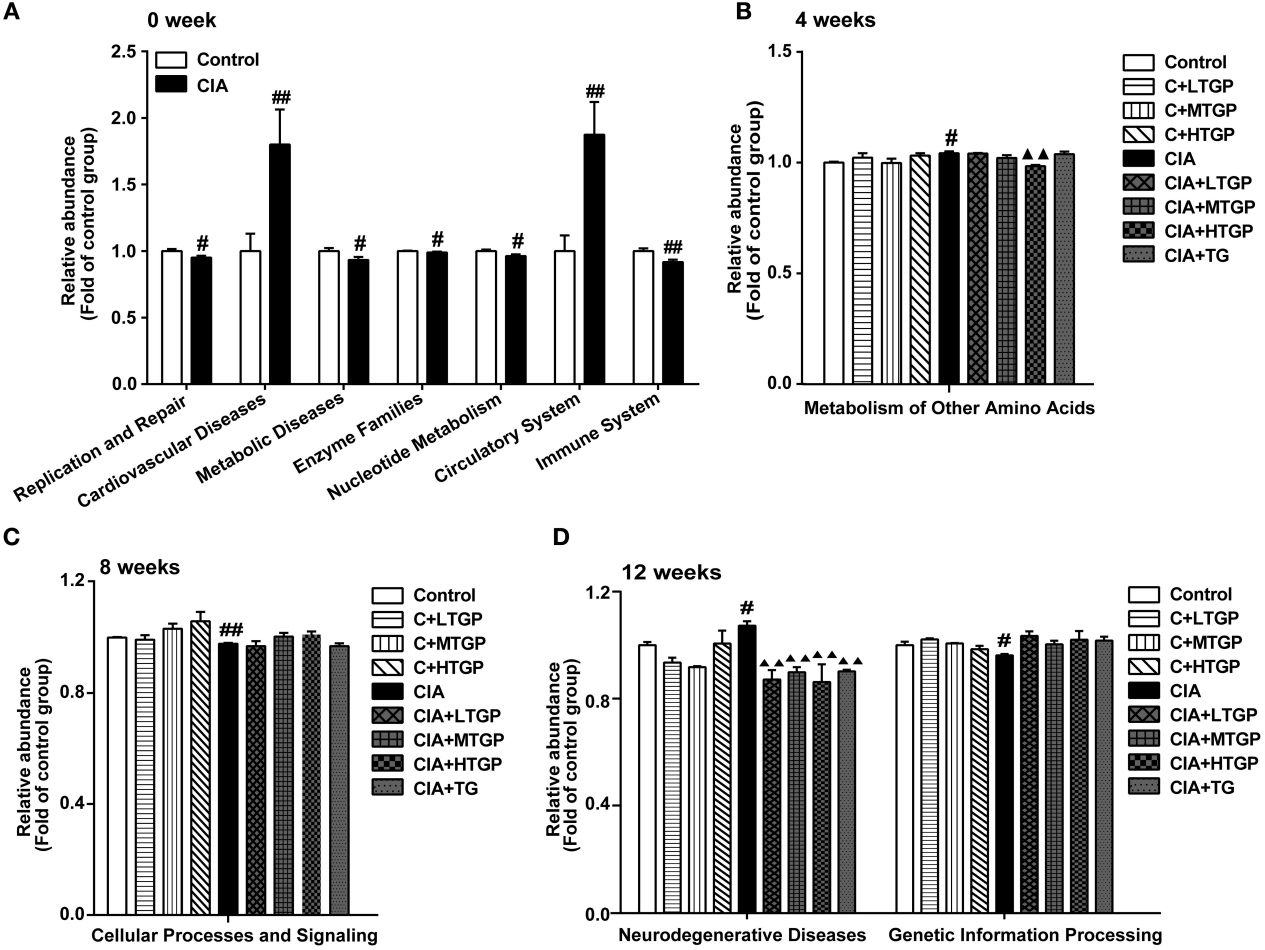
PICRUSt predicted the distribution of KEGG secondary metabolic pathways. **(A–D)** The relative abundance of KEGG secondary metabolic pathways at 0, 4, 8, 12 weeks, respectively. Control is the normal control group treated with just normal saline. C+LTGP, C+MTGP and C+HTGP are the control groups treated with 158, 474, and 948 mg/kg TGP, respectively. CIA is the collagen-induced arthritis group treated with just normal saline. CIA+ LTGP, C+MTGP and C+HTGP are the collagen-induced arthritis groups treated with 158, 474, and 948 mg/kg TGP, respectively. CIA+TG is the collagen-induced arthritis group treated with 7.89 mg/kg TG. Data indicate mean ± SEM. ^##^*P* <0.01, ^#^*P* <0.05 vs. Control; ^▴▴^*P* <0.01, ^▴^*P* <0.05 vs. CIA, indicating significant differences.

Next, PICRUSt tool was used to predict the functional profiles of gut microbiota (Figure 4). With the predicted metagenome, Kyoto Encyclopedia of Genes and Genomes (KEGG) pathway functions were categorized at level 2 using the PICRUSt. Compared to the Control group, the relative abundances of cardiovascular diseases (*P* <0.01), circulatory system (*P* <0.01), metabolism of other amino acids (*p* < 0.05), and neurodegenerative diseases (*p* < 0.05) were significantly higher in the CIA group. In contrast, replication and repair (*p* < 0.05), metabolic diseases (*p* < 0.05), enzyme families (*p* < 0.05), nucleotide metabolism (*p* < 0.05), immune system (*P* <0.01), cellular processes and signaling (*P* <0.01), and genetic information processing (*p* < 0.05) were significantly less abundant in the CIA group than in the Control group (Figures 4A–D). Thus, it was evident that the metabolic pathways of the gut microbiota in CIA rats differed from those in Control rats. Furthermore, after 4, 8, and 12 weeks of TGP intervention, TGP partially corrected the observed dysfunction in the gut microbiota. Compared to the CIA group, the relative abundance of metabolism for other amino acids (*P* <0.01) was significantly decreased in the CIA+HTGP group, and the relative abundance of neurodegenerative diseases was significantly decreased in the CIA+LTGP (*P* <0.01), CIA+MTGP (*P* <0.01), CIA+HTGP (*P* <0.01), and CIA+TG (*P* <0.01) groups. These results indicated that CIA disrupted the metabolic activity of the gut microbiota and that the disordered metabolic activity of the gut microbiota had been repaired at least in part, after 4, 8, and 12 weeks of TGP intervention.

In summary, CIA rats exhibited a distinct structure in their gut microbial community compared with control rats. Dysbiosis and dysfunction of the gut microbiota in CIA rats was also repaired by TGP intervention at least in part.

### Effects of TGP Upon Body Weight and Thymus and Spleen Indices of CIA Rats

Body weights were standardized in all groups before experiment. As expected, the all CIA groups gained less body weight than the Control group at day 28. Compared to the Control group, body weight decreased in the CIA group (*P* <0.01) from day 28 to day 112. Compared to the CIA group, body weight decreased in the CIA+HTGP group (*P* <0.01) from day 56 to day 112, meanwhile, body weight decreased in the CIA+MTGP group (*p* < 0.05) at day 112. As well as, compared to the Control group, the body weight decreased in the C+HTGP group (*P* <0.01) from day 84 to day 112 (Table 1).

**Table 1:** The influence of TGP on the body weight of CIA rats ($\bar{x}\;\pm \;SEM$).

**Group**	**N**	**Day 0**	**Day 28**	**Day 56**	**Day 84**	**Day 112**
Control	9	173 ± 2.32	394 ± 6.07	505 ± 9.46	569 ± 12.74	609 ± 15.04
C+LTGP	9	172 ± 1.99	396 ± 13.06	490 ± 21.42	545 ± 25.73	580 ± 28.95
C+MTGP	9	175 ± 2.79	400 ± 11.95	481 ± 18.97	537 ± 20.31	567 ± 21.66
C+HTGP	9	172 ± 2.37	417 ± 13.03	465 ± 9.42	417 ± 13.12^##^	331 ± 11.94^##^
CIA	10	172 ± 2.58	313 ± 11.23^##^	428 ± 15.29^##^	500 ± 19.70^##^	537 ± 18.27^##^
CIA +LTGP	10	176 ± 2.47	313 ± 14.15	395 ± 14.12	468 ± 17.25	507 ± 22.00
CIA +MTGP	10	171 ± 2.77	297 ± 8.44	394 ± 14.24	452 ± 18.61	469 ± 18.67^▴^
CIA +HTGP	10	171 ± 1.48	304 ± 11.72	324 ± 15.05^▴▴^	343 ± 16.18^▴▴^	286 ± 14.33^▴▴^
CIA +TG	10	171 ± 2.09	310 ± 14.94	411 ± 14.37	463 ± 13.91	502 ± 13.55

*Datas were expressed as mean ± SEM, day 0 (before inducing CIA), day 28 (before gavage), day 56 (4 weeks of intragastric administration), day 84 (8 weeks of intragastric administration), day 112 (12 weeks of intragastric administration)*.

## *P <0.01, ^#^P <0.05 vs. Control*;

▴▴ *P <0.01*,

▴ *P <0.05 vs. CIA, indicating significant differences*.

**Figure 5:**
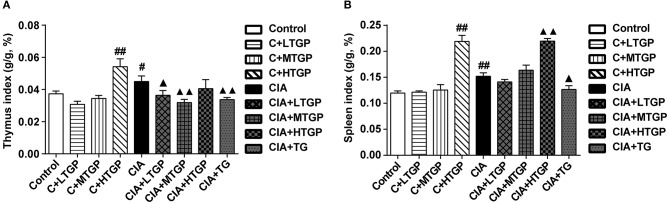
Effect of TGP on thymus and spleen indexes of CIA rats. **(A,B)** Statistical plot of thymus and spleen indexes, respectively. Control is the normal control group treated with just normal saline. C+LTGP, C+MTGP, and C+HTGP are the control groups treated with 158, 474, and 948 mg/kg TGP, respectively. CIA is the collagen-induced arthritis group treated with just normal saline. CIA+ LTGP, C+MTGP and C+HTGP are the collagen-induced arthritis groups treated with 158, 474, and 948 mg/kg TGP, respectively. CIA+TG is the collagen-induced arthritis group treated with 7.89 mg/kg TG. Data indicate mean ± SEM. ^##^*P* <0.01, ^#^*P* <0.05 vs. Control; ^▴▴^*P* <0.01, ^▴^*P* <0.05 vs. CIA, indicating significant differences.

Thymus index (*p* < 0.05) and spleen index (*P* <0.01) were higher in the CIA group than in the Control group. However, thymus index was lower in CIA+LTGP (*p* < 0.05), CIA+MTGP (*P* <0.01), and CIA+TG (*P* <0.01) groups than in the CIA group, meanwhile, spleen index was lower in CIA+TG group (*p* < 0.05) but higher in CIA+HTGP group (*P* <0.01) than in the CIA group. Compared to the Control group, thymus index (*P* <0.01) and spleen index (*P* <0.01) increased in the C+HTGP group (Figure 5).The above results suggested that different doses of TGP exhibited a remarkable influence on body weight and organ index in control and CIA rats.

### Effects of TGP Upon the Histology of CIA Rats

**Figure 6:**
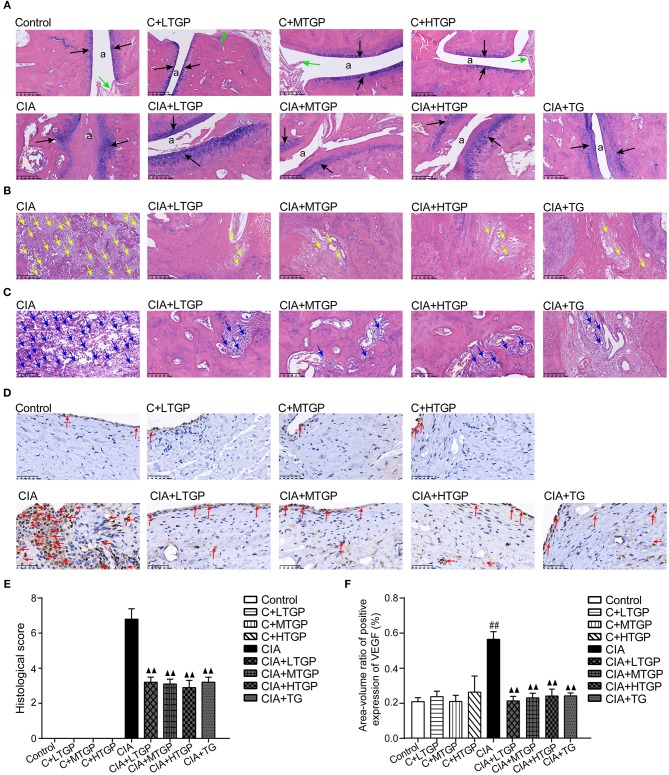
Effect of TGP on histologic assessment of CIA rats. **(A)** Representative sections of ankle joint stained with (H&E).The position of “a” represents the joint capsule, the black arrow indicates the articular cartilage, and the green arrow indicates the normal synovial cells. Magnifcation, 100×. **(B)** Representative sections of ankle joint stained with (H&E). The yellow arrow indicates the synovial hyperplasia. Magnifcation, 100×. **(C)** Representative sections of ankle joint stained with (H&E). The blue arrow indicates the inflammatory cells. Magnifcation, 200×. **(D)** Immunohistochemistry was performed to detect immunoreactivity for VEGF in synovium. The red arrow indicates the VEGF. Magnifcation, 400×. **(E)** Histological score was calculated. **(F)** The expression level of VEGF was detected. Control is the control group treated with just normal saline. C+LTGP, C+MTGP, and C+HTGP are the control groups, respectively, treated with 158, 474, and 948 mg/kg TGP. CIA is the collagen-induced arthritis group treated with just normal saline. CIA+ LTGP, C+MTGP and C+HTGP are the collagen-induced arthritis groups, respectively, treated with 158, 474, and 948 mg/kg TGP. CIA+TG is the collagen-induced arthritis group treated with 7.89 mg/kg TG. Data indicate mean ± SEM. ^##^*P* <0.01, ^#^*P* <0.05 vs. Control; ^▴▴^ *P* <0.01, ^▴^ *P* <0.05 vs. CIA, indicating significant differences.

Histopathological evaluation revealed pathological changes in the ankle joint. In the Control groups, the articular surface was covered with hyaline cartilage, the joint capsule was smooth, the synovial cells were continuous and the structure was clear. No pathological changes were observed in the Control rats, such as synovial swelling, tissue necrosis or inflammatory cell infiltration. However, in the CIA group, the cartilage on the articular surface was eroded, the joint capsule was narrow, and the synovial tissue had proliferated; this was accompanied by a large amount of inflammatory cell infiltration. After 12 weeks of administration, it was evident that TGP (*P* <0.01) and TG (*P* <0.01) had protected the articular cartilage, inhibited synovial tissue hyperplasia, and reduced inflammatory cell infiltration, at least to a certain extent (Figures 6A–C,E).

Expression of VEGF in the synovium was investigated by immunohistochemical staining and image analysis. Compared to the Control group, the expression of VEGF in the CIA group had increased significantly (*P* <0.01) although there were no significant changes in the C+LTGP, C+MTGP, and C+HTGP groups. However, compared to the CIA group, the expression of VEGF in the CIA+LTGP (*P* <0.01), CIA+MTGP (*P* <0.01), CIA+HTGP (*P* <0.01), and CIA+TGP (*P* <0.01) groups had decreased significantly. In conclusion, TGP administration had no effect on the expression of VEGF in control rats but significantly improved VEGF levels in CIA rats. TG also played a significant role in reducing the expression of VEGF in CIA rats (Figures 6D,F).

### Effect of TGP on T Cell Subsets in the Peripheral Blood Mononuclear Cells of CIA Rats

**Figure 7:**
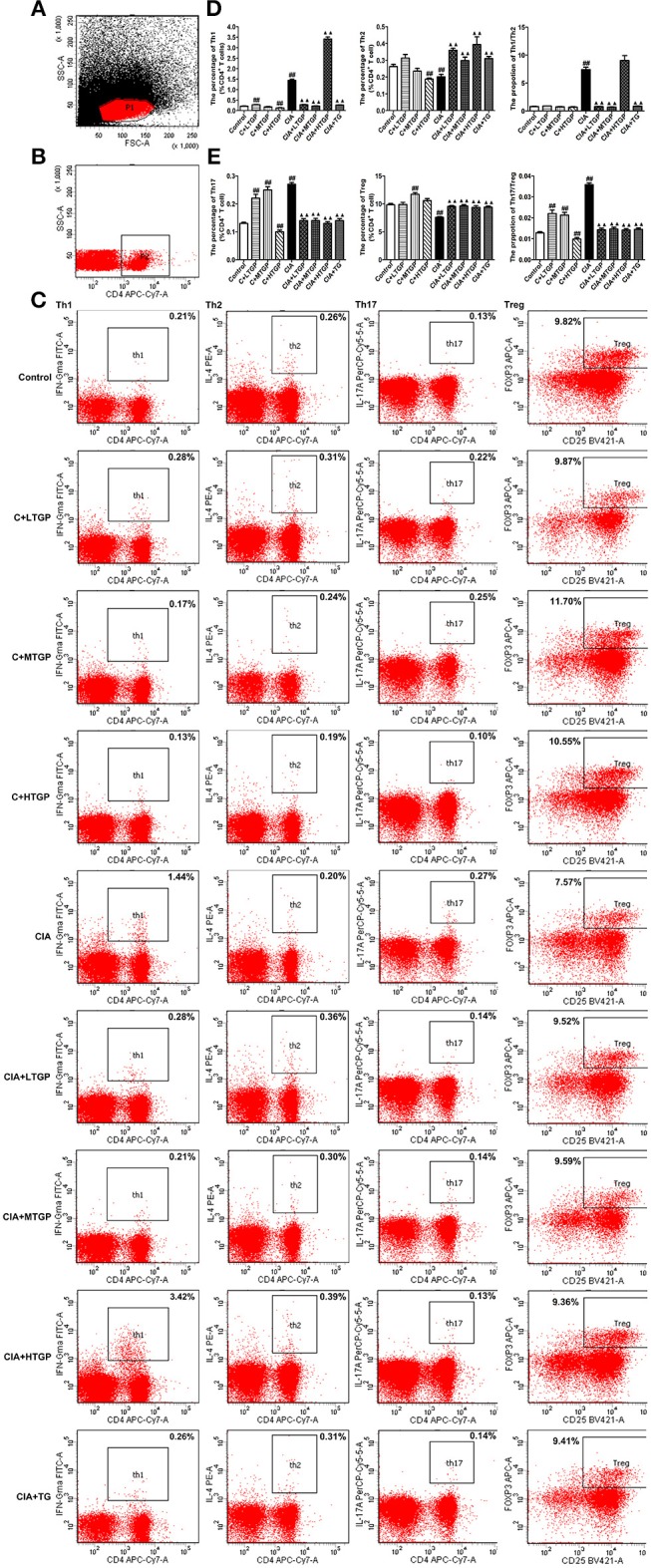
Effect of TGP on T cell subsets in peripheral blood mononuclear cells of CIA rats. **(A)** Lymphocytes. FSC/SSC were gated. **(B)** CD4^+^ T lymphocytes. SSC/CD4 were gated. **(C)** Intracellular expressions of IFN-γ, IL-4, IL-17A, Foxp3 on CD3^+^ CD4^+^ T cells were gated. **(D)** Percentages of Th1, Th2 cells and Th1/Th2 ratio were analyzed. **(E)** Percentages of Th17 and Treg cells and Th17/Treg ratio were analyzed. Control is the normal control group treated with just normal saline. C+LTGP, C+MTGP and C+HTGP are the control groups treated with 158, 474, and 948 mg/kg TGP, respectively. CIA is the collagen-induced arthritis group treated with just normal saline. CIA+ LTGP, C+MTGP, and C+HTGP are the collagen-induced arthritis groups treated with 158, 474, and 948 mg/kg TGP, respectively. CIA+TG is the collagen-induced arthritis group treated with 7.89 mg/kg TG. Data indicate mean ± SEM. ^##^*P* <0.01, ^#^*P* <0.05 vs. Control; ^▴▴^ *P* <0.01, ^▴^ *P* <0.05 vs. CIA, indicating significant differences.

To determine the proportion (in %) of Th1, Th2, Th17, and Treg cells after TGP intervention, flow cytometry was conducted. Representative flow cytometry results for the four T cell subsets, in each of the different experimental groups, are shown in Figures 7A–C.

Compared to the Control group, the proportion of Th1 cells (*P* <0.01) and the ratio of Th1/Th2 cells (*P* <0.01) increased significantly in the CIA group; the proportion of Th2 cells, however, decreased significantly (*P* <0.01). In contrast, compared to the CIA group, the proportion of Th1 cells decreased significantly in the CIA+LTGP (*P* <0.01), CIA+MTGP (*P* <0.01), and CIA+TG (*P* <0.01) groups, but increased significantly in the CIA+HTGP group (*P* <0.01). The proportion of Th2 cells increased significantly in the CIA+LTGP (*P* <0.01), CIA+MTGP (*P* <0.01), CIA+HTGP (*P* <0.01), and CIA+TG (*P* <0.01) groups, while the ratio of Th1/Th2 cells decreased significantly in the CIA+LTGP (*P* <0.01), CIA+MTGP (*P* <0.01) and CIA+TG (*P* <0.01) groups. Meanwhile, compared to the Control group, the proportion of Th1 cells increased significantly in the C+LTGP (*P* <0.01) group, while the proportion of Th1 cells (*P* <0.01) and Th2 cells (*P* <0.01) decreased significantly in the C+HTGP group (Figure 7D).

Compared to the Control group, the proportion of Th17 cells (*P* <0.01) and the ratio of Th17/Treg cells (*P* <0.01) increased significantly in the CIA group, while the proportion of Treg cells (*P* <0.01) decreased significantly. In contrast, compared to the CIA group, the proportion of Th17 cells decreased significantly in the CIA+LTGP (*P* <0.01), CIA+MTGP (*P* <0.01), CIA+HTGP (*P* <0.01), and CIA+TG (*P* <0.01) groups, the proportion of Treg cells increased significantly in the CIA+LTGP (*P* <0.01), CIA+MTGP (*P* <0.01), CIA+HTGP (*P* <0.01), and CIA+TG (*P* <0.01) groups and the ratio of Th17/Treg cells decreased significantly in the CIA+LTGP (*P* <0.01), CIA+MTGP (*P* <0.01), CIA+HTGP (*P* <0.01) and CIA+TG (*P* <0.01) groups. Compared to the Control group, the proportion of Th17 cells increased significantly in the C+LTGP (*P* <0.01) group, the proportions of Th17 cells (*P* <0.01) and Treg cells (*P* <0.01) increased significantly in the C+MTGP group, and the proportion of Th17 cells (*P* <0.01) decreased in the C+HTGP group, the ratio of Th17/Treg increased significantly in the C+LTGP (*P* <0.01) and C+MTGP (*P* <0.01) groups but decreased significantly in the C+HTGP group (*P* <0.01) (Figure 7E). These results suggested that TGP reversed the evident imbalance in both Th1/Th2 and Th17/Treg in CIA rats, but had no influence upon Th1/Th2 and Th17/Treg in Control rats.

### Effect of TGP Upon Intestinal Cytokines in CIA Rats

**Figure 8:**
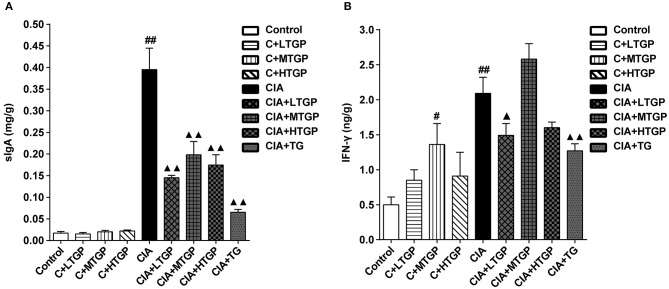
Effect of TGP on intestinal cytokines of CIA rats. **(A)** The levels of SIgA in contents of small intestine was estimated by ELISA. **(B)** The levels of IFN-γ in small intestine tissue was estimated by ELISA. Control is the normal control group treated with just normal saline. C+LTGP, C+MTGP, and C+HTGP are the control groups treated with 158, 474, and 948 mg/kg TGP, respectively. CIA is the collagen-induced arthritis group treated with just normal saline. CIA+LTGP, C+MTGP, and C+HTGP are the collagen-induced arthritis groups treated with 158, 474, and 948 mg/kg TGP, respectively. CIA+TG is the collagen-induced arthritis group treated with 7.89 mg/kg TG. Data indicate mean ± SEM. ^##^*P* <0.01, ^#^*P* <0.05 vs. Control; ^▴▴^ *P* <0.01, ^▴^*P* <0.05 vs. CIA, indicating significant differences.

The levels of SIgA in the contents of the small intestine and IFN-γ in tissues from the small intestine of rats were measured by specific ELISA kit after 12 weeks of intragastric TGP administration. Compared to the Control group, the levels of SIgA (*P* <0.01) and IFN-γ (*P* <0.01) were significantly higher in the CIA group. However, compared to the CIA group, the levels of SIgA were significantly lower in the CIA+LTGP (*P* <0.01), CIA+MTGP (*P* <0.01), CIA+HTGP (*P* <0.01), and CIA+TG (*P* <0.01) groups; furthermore, the levels of IFN-γ were significantly lower in the CIA+LTGP (*p* < 0.05) and CIA+TG (*P* <0.01) groups. Compared to the Control group, the levels of IFN-γ were significantly higher in the C+MTGP group (*p* < 0.05) (Figures 8A,B). These results suggested that the immune response of the intestinal mucosa was up-regulated in the arthritis state and that TGP and TG may have a regulatory effect upon the immune response of the intestinal mucosa.

## Discussion

The gut microbiota plays a crucial role in the maintenance of human health and is regarded as another “hidden organ” carrying the “second genome” of the human body. Under normal circumstances, there is a complex and delicate dynamic balance between the gut microbiota and the host (Janssen and Kersten, 2015). The current analyses identified significant differences in the microbial composition of CIA rats, as compared to control rats, after 0, 4, 8, and 12 weeks of TGP administration. Taxonomic analyses, carried out at these four time points, showed that the relative abundances of two phyla, four classes, four orders, four families, and four genera increased significantly in the CIA group, while the relative abundances of one phylum, one class, one order, three families, and seven genera decreased significantly. LEfSe analysis also carried out at these four time points, further showed that the relative abundances of two phyla, four classes, five orders, five families, and six genera were the highest in the CIA group, while that of one family and one genera were the highest in the Control group. Previous research had identified that the structure of such microorganisms changed significantly with the progression of arthritis and autoimmune diseases. For example, with regards to immune arthritis, the abundance of Proteobacteria was reported to increase during the immune-priming phase of CIA (Rogier et al., 2017). Mice that were sensitive to CIA showed an enrichment of OTUs affiliated with *Lactobacillus* as the dominant genus prior to the onset of arthritis (Liu et al., 2016). Actinobacteria is present only in patients with Juvenile Idiopathic Arthritis (JIA) (Tejesvi et al., 2016). The abundance of *Pseudomonas* has also been reported to increase significantly in subjects with reactive arthritis (ReA) (Manasson et al., 2018). With regards to other immune diseases, the abundance of *Akkermansia* was reported to increase significantly in untreated twins with multiple sclerosis (MS) (Berer et al., 2017). High levels of *Lactobacillus* have also been reported in patients with systemic sclerosis (SSc) (Patrone et al., 2017). In another study, the abundance of Actinobacteria was positively correlated with levels of serum malondialdehyde (MDA) in patients with systemic lupus erythematosus (SLE) (Gonzalez et al., 2017). Interestingly, higher concentrations of serum copper in SLE patients were also positively associated with levels of C reactive protein (CRP); levels of CRP were also associated with the proportion of Proteobacteria and Verrucomicrobia in feces (Gonzalez et al., 2017). Lipopolysaccharide, a component of the cell wall in Proteobacteria is a known inducer of lupus in mice (Mu et al., 2017); this may represent a potential mechanism by which Proteobacteria are associated with the onset of autoimmune diseases. These previous results are consistent with our present findings, which indicated that an increased abundance of Proteobacteria, *Lactococcus*, Actinobacteria, *Pseudomonas, Akkermansia*, and Verrucomicrobia are associated with the onset of disease.

It also has been reported that some of these microorganisms may be associated with resistance to certain immune diseases. For example, levels of Prevotellaceae were relatively low in patients with ankylosing spondylitis (AS) (Costello et al., 2015). Patients with uveitis (UVT) were also known to exhibit a low abundance of Ruminococcaceae (Kalyana Chakravarthy et al., 2018). Previous research also showed that donors with abundant *unclassified_Ruminococcaceae* were more likely to induce remission when fecal microbiota transplantation was used to treat ulcerative colitis (Kump et al., 2018). Interestingly, rats that had been infected with bacteria as newborns have been shown to exhibit life-long vulnerabilities in terms of cognitive dysfunction, while neonatal infection with *Escherichia coli* caused a reduction in genera belonging to the Tenericutes phylum (Williamson et al., 2016). Oral exposure to cadmium (Cd) can also induce various adverse health effects in both humans and animals, and can also reduce the relative abundance of Mollicutes (Qixiao et al., 2017). These earlier results are consistent with our current findings, which suggest that Prevotellaceae, Ruminococcaceae, *unclassified_Ruminococcaceae*, Tenericutes, and Mollicutes may be associated with disease resistance.

After 4, 8, and 12 weeks of TGP intervention, taxonomic analyses showed that the relative abundances of one phylum, one class, one order, one family and three genera increased following the administration of TGP. LEfSe analysis further showed that the relative abundances of one class, one order, two families and five genera were the highest in CIA rats that had been treated with TGP. Interestingly, exposure to copper (Cu) and lead (Pb) has been reported to result in a marked reduction in *Ruminococcaceae_UCG-014* and *Oscillibacter*, respectively (Qixiao et al., 2017). Previous research showed that the relative abundance of *Clostridium* was reduced in patients with SSc and that *Clostridium* acts as a beneficial commensal genus to prevent gastrointestinal dysfunction in such patients (Patrone et al., 2017; Volkmann, 2017). Other studies found that Resveratrol could improve glucose homeostasis in obese mice, and cause an increase in the relative abundance of *Parabacteroides* (Sung et al., 2017). These earlier reports are consistent with our current findings, which indicate that TGP intervention increased the relative abundances of the beneficial symbiotic bacteria *Ruminococcaceae_UCG-014, Oscillibacter*, and *Parabacteroides*. TG intervention led to an increase in the relative abundance of *Clostridium* as a beneficial symbiotic bacterium.

In summary, we detected significant differences in the structure of the gut microbiota between Control and CIA rats at 0, 4, 8, and 12 weeks. However, after TGP intervention, the microbial structure of the gut changed in CIA rats. In addition, we also found that the difference between the CIA group and the Control group reduced gradually over time. This observation indicated that this microbiological system was complex and may possess a powerful ability for self-repair; previous work reported that the gut microbiota can maintain and repair itself via self-replication (Backhed et al., 2005). However, in combination with the ameliorative effects of TGP on CIA rats, repairs made to the gut microbiota without drug intervention appears to have no effect upon treatment. This indicated that once CIA is successfully established, it is difficult to rely upon the self-repairing ability of the bacteria to ameliorate disease symptoms; consequently, drug intervention must be carried out as early as possible. Nevertheless, we also found that irrespective of the difference between the CIA and control groups, the administration of TGP could not only correct 78% of the differential taxonomic composition, but also increase the relative abundances of certain forms of beneficial symbiotic bacteria, thus ameliorating the intestinal micro-ecological environment in CIA rats. Moreover, we found that the metabolic activities of gut microbiota could contribute to the pathogenesis of CIA. We used PICRUSt to investigate functional disorders in the gut microbiota of CIA rats before and during TGP intervention; dysfunction was observed in the gut microbiota of CIA rats over time, but alterations in the microbial function of CIA rats were partly corrected by TGP intervention.

Body weight plays a role in assessing the growth state. It can be found that three different dosages of TGP can cause the soft stools in rats, consistent with the description of the adverse reactions of “occasion of soft stools” in the drug insert. It was speculated that TGP may have a dose-dependent effect on the body weight. Thymus and spleen indexes can reflect the immune activity (Xia et al., 2018). The experimental results showed that the thymus and spleen indexes in CIA group were significantly increased compared to the Control group, it indicated that the CIA rats were in a hyperthyroid state. After administration of TGP or TG, the thymus and spleen indexes in some groups were significantly reduced compared to the CIA group. The above phenomena indicated that TGP and TG may have immunosuppressive effects on the central and peripheral lymphoid organs of CIA rats, then induce immune tolerance.

Previous studies have shown that VEGF can promote vascular proliferation, enhance vascular permeability and that VEGF levels are closely related to the progression of RA (Gurzu et al., 2017). In the present study, low levels of VEGF expression was found in the synovium of Control rats, suggesting that this factor plays a role in maintaining the normal function of the synovium. Synoviocytes in the CIA rats showed active proliferation and exhibited significantly higher levels of VEGF. TGP and TG significantly inhibited the expression of VEGF in CIA rats, indicating that TGP and TG may inhibit neovascularization and the abnormal proliferation of synoviocytes by regulating VEGF levels.

Previous studies suggest that an imbalance in Th1/Th2 and Th17/Treg cells plays an important role in the pathogenesis and progression of RA (Lina et al., 2011). Indeed, some studies suggest that the regulation of Th1/Th2 and Th17/Treg balance is a useful method with which to treat RA (Wang et al., 2012; Ma, 2013). In the present study, the balance of T cell subsets in the peripheral blood mononuclear cells was inclined to Th1 and Th17 cells in the CIA group; after intervention with TGP and TG, the ratios of Th1 and Th17 cells in CIA rats were down-regulated, while the ratios of Th2 and Treg cells were up-regulated; we believe that these changes were related to the evident improvement in CIA symptoms following TGP and TG administration. Immunoglobulin A (IgA) is the most abundant and heterogeneous immunoglobulin in humans (Kerr, 1990). There are two forms of IgA, named after their source and location: serum IgA and secretory IgA. Serum IgA is mainly monomeric and produced by B lymphocytes in the bone marrow and some lymphoid organs. In mammals, however, most IgA is found in mucosal secretions. Secretory IgA (SIgA) consists of IgA dimers and a polymeric immunoglobulin receptor-derived polypeptide (Phalipon et al., 2002). SIgA is the most abundant antibody in the intestinal mucosa (Cerutti et al., 2011; Corthesy, 2013) and is derived from two sources (Kerr, 2000; Macpherson et al., 2000). Approximately 75% of SIgA originates from B2 lymphocytes in organized germinal centers of mucosal lymphoid tissues, such as Peyer's patches. This form of SIgA production is dependent upon T lymphocytes. A second source, contributing to approximately 25% of SIgA production, is the B1 lymphocytes which develop in the peritoneal cavity and are widely distributed in the intestinal lamina propria. This form of SIgA may represent a primitive source of SIgA that is independent of T lymphocytes and recognizes commensal bacteria. SIgA plays an important role in the immune defense function of the intestinal mucosa by preventing intestinal antigens, microorganisms and other foreign antigens from adhering to and invading the surface of the intestinal mucosa, thereby preventing the occurrence of intestinal infection (Wershil and Furuta, 2008; Cerutti et al., 2011). The present study showed that the level of SIgA in the contents of small intestine in the CIA group had increased significantly compared to the Control group, suggesting that the immune response of the intestinal mucosa in CIA rats was up-regulated. TGP and TG were able to significantly down-regulate the secretion of SIgA, suggesting that these two drugs exert a regulatory effect upon the immune response of the intestinal mucosa. IFN-γ is found in large amounts in the small intestine and is mainly produced by CD4^+^ T cells; IFN-γ plays an important role in promoting B cell differentiation and the secretion of SIgA (Kjerrulf et al., 1997). The current data showed that the increased levels of IFN-γ in the small intestine of CIA rats may contribute to the increased secretion of SIgA. TGP and TG were shown to reduce the levels of IFN-γ in the small intestine, which is consistent with the hypothesis that these factors down-regulate the secretion of SIgA.

In this study, the effect of long-term TGP administration on CIA rats was comprehensively evaluated from organ index, ankle joint morphology, synovial VEGF content, peripheral blood immunity, and intestinal cytokines. The results suggested that TGP may inhibit angiogenesis and the proliferation of synoviocytes by regulating levels of VEGF. TGP also maintained the balance of T cell subsets in CIA rats by down-regulating the levels of Th1 cells and Th17 cells, and by up-regulating the levels of Th2 cells and Treg cells. TGP also inhibited levels of the intestinal cytokines SIgA and IFN-γ. TGP may induce autoimmune tolerance and inhibit inflammatory response. We also identified a number of key microbial genera that show association with TGP treatment and provide evidence to demonstrate that the gut microbiota may play a role in the TGP-mediated amelioration of CIA symptoms. Via these effects, TGP protected the ankle joint and the structure of the gut microbiota in CIA rats. Whether TGP ameliorates CIA via interaction between the gut microbiota and intestinal mucosal immunity remains unknown and deserves further research attention.

## Data Availability

The 16S rRNA gene sequence data (accession number: PRJNA507839, SRP171806) have been deposited in the NCBI's Sequence Read Archive database. Raw data for Table 1 and Figures 5–8 have been included in Supplementary Data Sheet 1. All datasets analyzed during the current study are available from the corresponding author on reasonable request.

## Ethics Statement

Rats were housed under specific pathogen-free (SPF) conditions at the Laboratory Animal Center of Capital Medical University, Beijing, China, with 55–60% humidity and 12-h light/dark cycle, at 22–24°C. Rats were given free access to food and water that were sterilized. Animal protocols were approved by the Capital Medical University Animal Experiments and Experimental Animals Management Committee (Beijing, China, Ethical Approval Number: AEEI-2014-128), and all experimental procedures conformed to the National Institutes of Health Guide for the Care and Use of Laboratory Animals. All efforts were made to minimize the number of animals used.

## Author Contributions

JP and MG conceived and designed the experiments, drafted, and revised the manuscript. JP, XL, KX, YX, LG, YH, XD, YL, and ZL performed the experiments. XL, RH, and SW analyzed the data and drafted the figures. WC performed the literature search. MG was the corresponding author. All authors gave final approval for publication.

### Conflict of Interest Statement

The authors declare that the research was conducted in the absence of any commercial or financial relationships that could be construed as a potential conflict of interest.

## Abbreviations

AI: Arthritis index
ANOSIM: analysis of similarities
AS: ankylosing spondylitis
Cd: cadmium
CHB: chronic hepatitis B
Control: normal control group treated with just normal saline
C+HTGP: control group treated with 948 mg/kg TGP
C+LTGP: control group treated with 158 mg/kg TGP
C+MTGP: control group treated with 474 mg/kg TGP
CIA: collagen-induced arthritis
CIA: collagen-induced arthritis group treated with just normal saline
CIA+HTGP: collagen-induced arthritis group treated with 948 mg/kg TGP
CIA+LTGP: collagen-induced arthritis group treated with 158 mg/kg TGP
CIA+MTGP: collagen-induced arthritis group treated with 474 mg/kg TGP
CIA+TG: collagen-induced arthritis group treated with 7.89 mg/kg tripterygium glycosides
CRP: C reactive protein
CTM: Chinese traditional herbal medicine
Cu: copper
DAB, 3: 3′-diaminobenzidine
IFN-γ: Interferon-γ
JIA: Juvenile Idiopathic Arthritis
KEGG: Kyoto Encyclopedia of Genes and Genomes
LEfSe: linear discriminant analysis effect size method
MDA: malondialdehyde
MS: multiple sclerosis
NORA: new-onset untreated RA
Pb: lead
PICRUSt: Phylogenetic Investigation of Communities by Reconstructing Unobserved States
PLS-DA: partial least squares discriminant analysis
RA: Rheumatoid arthritis
ReA: reactive arthritis
SIgA: Secretory immunoglobulin A
SLE: systemic lupus erythematosus
SSc: systemic sclerosis
TG: tripterygium glycosides
TGP: total glucosides of paeony
UVT: uveitis
VEGF: vascular endothelial growth factor.
