# Supplementary material for: Dynamic Alterations in the Gut Microbiota of Collagen-Induced Arthritis Rats Following the Prolonged Administration of Total Glucosides of Paeony
Source: Front Cell Infect Microbiol. 2019 Jun 12;9:204. doi: 10.3389/fcimb.2019.00204 (PMC6581682; doi:10.3389/fcimb.2019.00204)
Supplement: Supplementary file 2 [file Data_Sheet_2.PDF]

Supplementary Material

Dynamic alterations in the gut microbiota of collagen-induced arthritis rats following the prolonged administration of total glucosides of paeony

Jine Peng, Xuran Lu, Kaili Xie, Yongsong Xu, Rui He, Li Guo, Yaxin Han, Sha Wu, Xuerong Dong, Yun Lu, Zhengyue Liu, Wei Cao, Muxin Gong*

*** Correspondence: Muxin Gong:** gongmuxin@126.com

1 Rarefaction curve of OTU level for Alpha Diversity Analysis

At 0 week, a total of 970012 effective sequences of 28 samples were generated with an average of 34643±992. At 4 weeks, a total of 1680214 effective sequences of 28 samples were generated with an average of 60008±1668. At 8 weeks, a total of 1072387 effective sequences of 27 samples were generated with an average of 39718±1257. At 12 weeks, a total of 1287954 effective sequences of 27 samples were generated with an average of 47702±453. Chao1 index and Shannon index analyses indicated that the sequencing depth rare covered new phylotypes and most of the diversity **(Supp.Fig.1A-D)**.

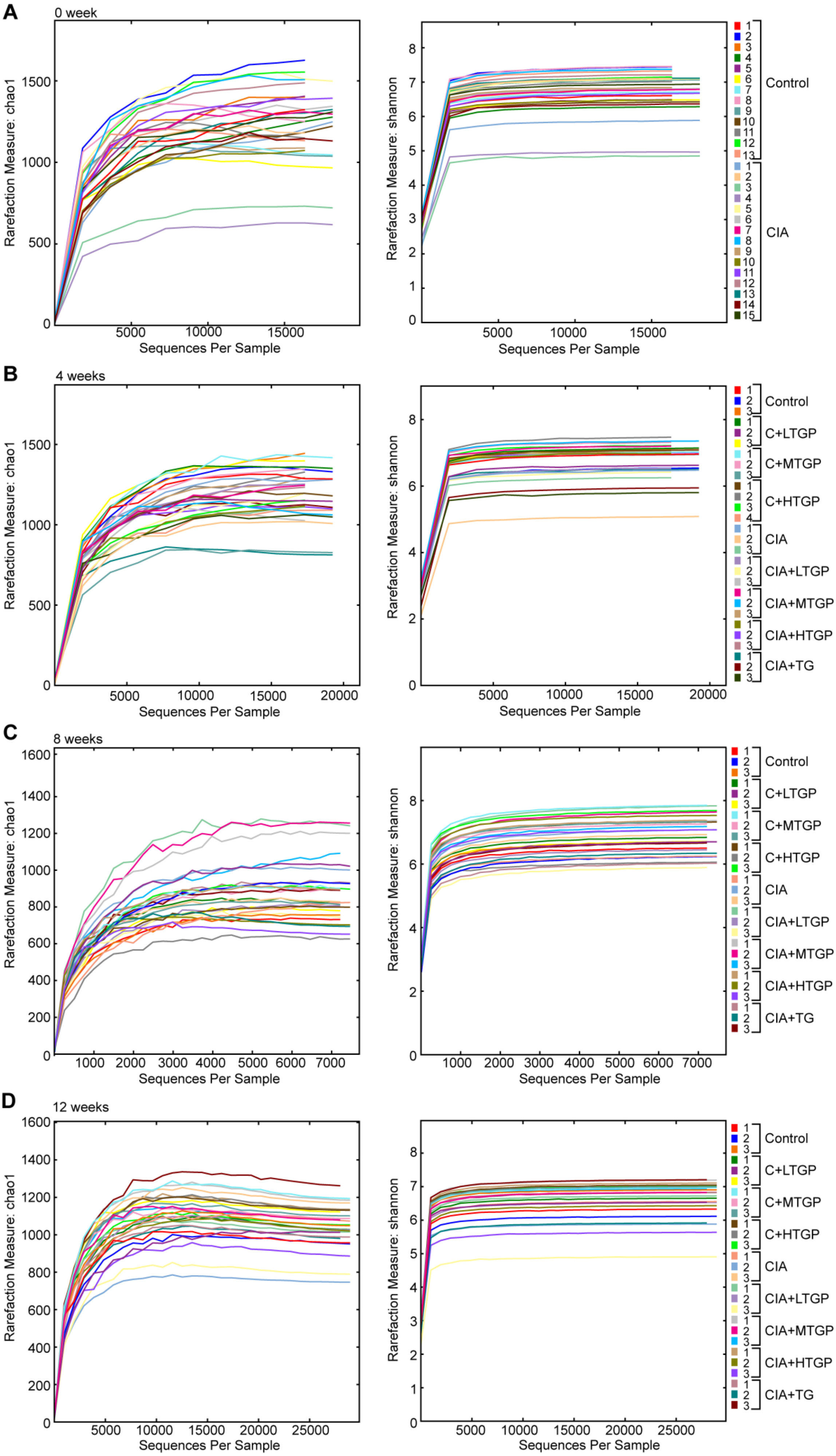
A
0 week
Rarefaction Measure: chao1
Sequences Per Sample
Rarefaction Measure: shannon
Sequences Per Sample
Control
CIA
B
4 weeks
Rarefaction Measure: chao1
Sequences Per Sample
Rarefaction Measure: shannon
Sequences Per Sample
Control
C+LTGP
C+MTGP
C+HTGP
CIA
CIA+LTGP
CIA+MTGP
CIA+HTGP
CIA+TG
C
8 weeks
Rarefaction Measure: chao1
Sequences Per Sample
Rarefaction Measure: shannon
Sequences Per Sample
Control
C+LTGP
C+MTGP
C+HTGP
CIA
CIA+LTGP
CIA+MTGP
CIA+HTGP
CIA+TG
D
12 weeks
Rarefaction Measure: chao1
Sequences Per Sample
Rarefaction Measure: shannon
Sequences Per Sample
Control
C+LTGP
C+MTGP
C+HTGP
CIA
CIA+LTGP
CIA+MTGP
CIA+HTGP
CIA+TG

Supplementary Figure 1. Rarefaction curve of OTU level for Alpha Diversity Analysis. (A, B, C, D) The diagram of Shannon curve and Chao1 curve at 0, 4, 8, 12 weeks, respectively. Control is the normal control group treated with just normal saline. C+LTGP, C+MTGP and C+HTGP are the control groups respectively treated with 158 mg/kg, 474 mg/kg, 948 mg/kg TGP. CIA is the collagen-induced arthritis group treated with just normal saline. CIA+LTGP, C+MTGP and C+HTGP are the collagen-induced arthritis groups respectively treated with 158 mg/kg, 474 mg/kg, 948 mg/kg TGP. CIA+TG is the collagen-induced arthritis group treated with 7.89 mg/kg tripterygium glycosides.

2 Venn diagram analysis of common OTU

The temporal changes of microbial richness between groups were presented by Venn diagram **(Supp.Fig.2A-D)**. At 0 week, the Venn diagram showed that the total OTUs of Control group and CIA group were 3815 and 4015, respectively. At 4 weeks, the total OTUs of Control group and CIA group were 2016 and 1713, respectively. And more than one third of the observed OTUs in the Control group were undetected in the CIA group. At 8 weeks, the total OTUs of Control group and CIA group were 1307 and 1443, respectively. But about 44% of the observed OTUs in the CIA group were undetected in the Control group. At 12 weeks, the total OTUs of Control group and CIA group were 1788 and 1743, respectively. And about 48% of the observed OTUs in the Control group were undetected in the CIA group. So, it can be seen that CIA reduced the diversity of intestinal flora at most time points. At 0 and 8 weeks, the number of OTUs in the CIA group were more than that in the Control group, while at 4 and 12 weeks, the number of OTUs in the CIA group were less than that in the Control group. These suggested that the structure of gut microbiota may change periodically. Simultaneously, the number of OTUs in TGP-treated CIA groups were more than that in CIA group after 4, 8, 12 weeks of TGP intervention, which indicated TGP could increase the diversity of gut microbiota in CIA rats.

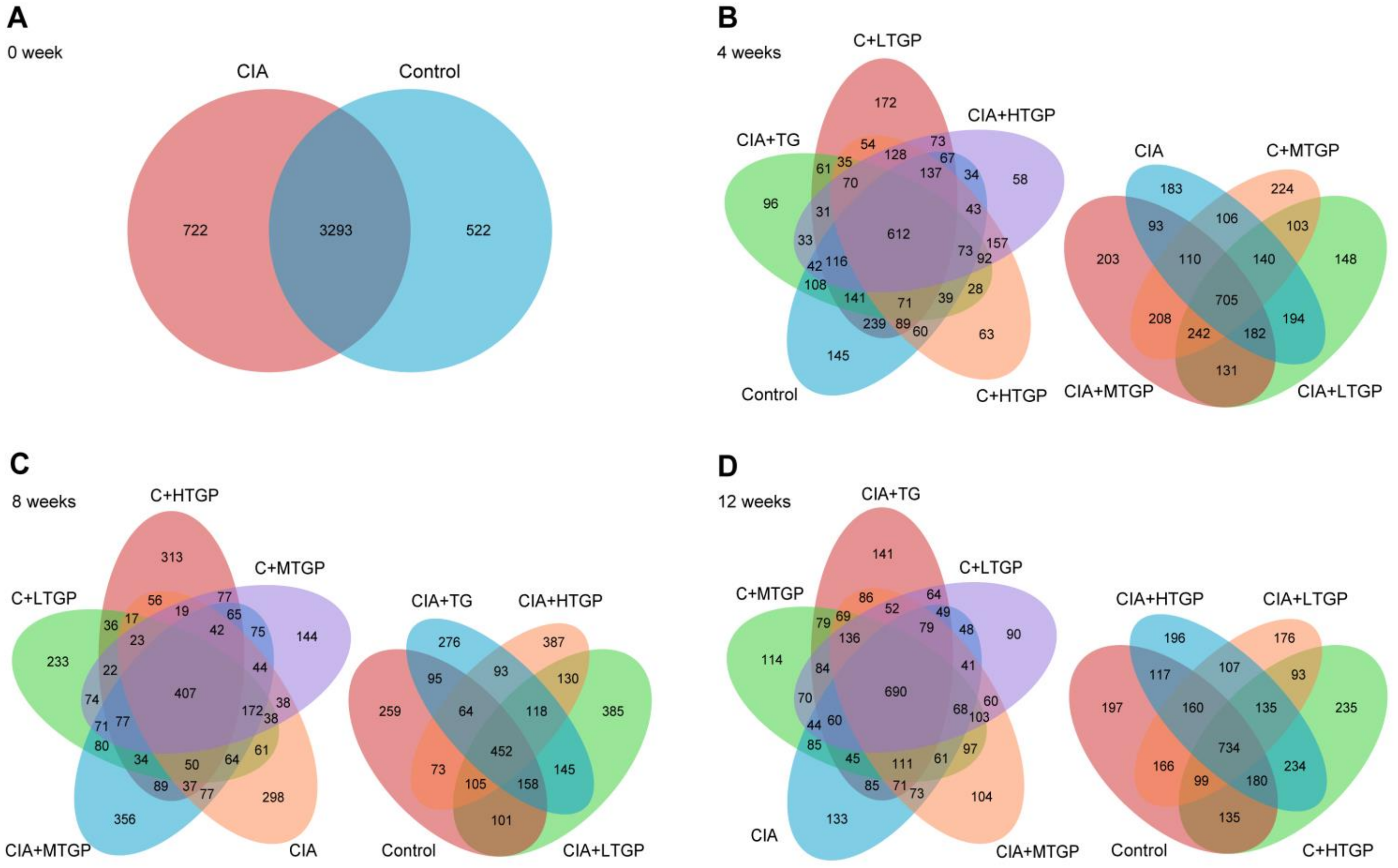

Supplementary Figure 2. Venn diagram analysis of common OTU. (A, B, C, D) The diagram of Venn diagram at 0, 4, 8, 12 weeks, respectively. Control is the normal control group treated with just normal saline. C+LTGP, C+MTGP and C+HTGP are the control groups respectively treated with 158 mg/kg, 474 mg/kg, 948 mg/kg TGP. CIA is the collagen-induced arthritis group treated with just normal saline. CIA+ LTGP, C+MTGP and C+HTGP are the collagen-induced arthritis groups respectively treated with 158 mg/kg, 474 mg/kg, 948 mg/kg TGP.

CIA+TG is the collagen-induced arthritis group treated with 7.89 mg/kg tripterygium glycosides.

3 PLS-DA revealed the similarity of gut microbial community

To measure the extent of the similarity of gut microbial communities, ANOSIM (analysis of similarities) of the unweighted UniFrac distance matrix and PLS-DA (partial least squares discriminant analysis) were performed. At 0, 4, 8, 12 weeks, the results of ANOSIM showed ($R=0.2827$, $P=0.001$), ($R=0.6830$, $P=0.001$), ($R=0.6029$, $P=0.001$) and ($R=0.3601$, $P=0.002$) respectively and revealed apparent separation in gut microbial structure of each group. At 0 week, the PLS-DA plot indicated obvious separation in gut microbial structure between the Control group and CIA group along PLS1 and PLS2 axes **(Supp.Fig.3A)**. At 4 weeks, there was obvious separation between the Control group and CIA group. Moreover, CIA+LTGP, CIA+MTGP and CIA+HTGP groups moved farther and farther away from the CIA group along the counter-clockwise direction, meanwhile, C+LTGP, C+MTGP and C+HTGP groups moved farther and farther away from the Control group along the counter-clockwise direction **(Supp.Fig.3B)**. At 8 weeks, the PLS-DA plot didn't indicate apparent separation in gut microbial structure between the Control group and CIA group. But CIA+LTGP, CIA+MTGP and CIA+HTGP groups moved farther and farther away from the CIA group along the clockwise direction, at the same time, C+LTGP, C+MTGP and C+HTGP groups moved farther and farther away from the Control group along the clockwise direction **(Supp.Fig.3C)**. At 12 weeks, there was clear separation between the Control group and CIA group. CIA+LTGP, CIA+MTGP and CIA+HTGP groups were separated from CIA group respectively, but not obviously. Simultaneously, C+LTGP, C+MTGP and C+HTGP groups moved from the Control group along the clockwise direction **(Supp.Fig.3D)**. Interestingly, all PLS-DA plots indicated no significant separation of gut microbial structure between CIA group and CIA+TG group. In summary, the results indicated the temporal changes of relationship among the gut microbial of each group, and the effect of TGP on gut microbiota was dose-dependent.

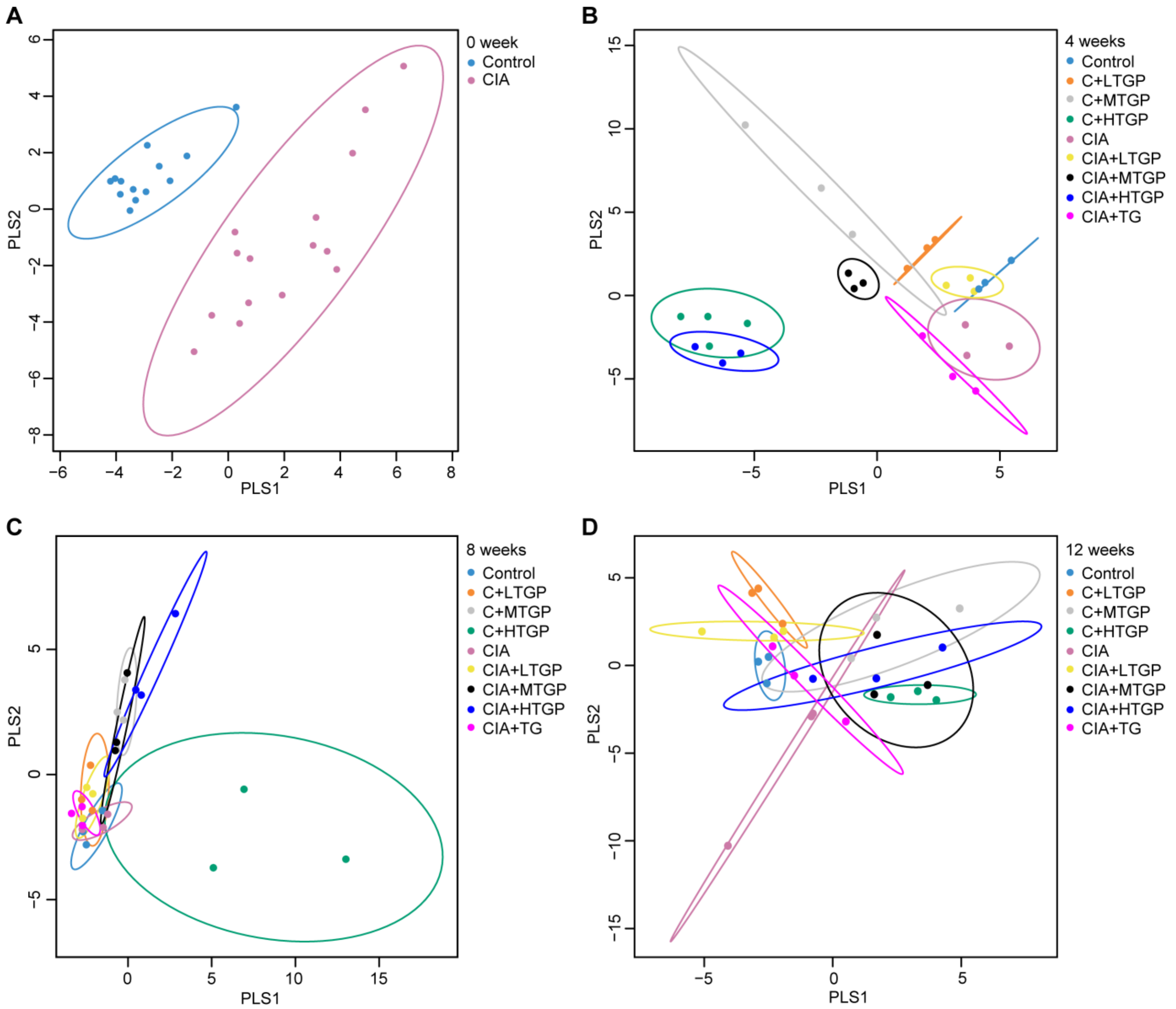

Supplementary Figure 3. PLS-DA revealed the similarity of gut microbial community. (A, B, C, D) Plots shown were generated using the PLS-DA at 0, 4, 8, 12 weeks, respectively. Control is the normal control group treated with just normal saline. C+LTGP, C+MTGP and C+HTGP are the control groups treated with 158 mg/kg, 474 mg/kg, 948 mg/kg TGP, respectively. CIA is the collagen-induced arthritis group treated with just normal saline. CIA+ LTGP, C+MTGP and C+HTGP are the collagen-induced arthritis groups treated with 158 mg/kg, 474 mg/kg, 948 mg/kg TGP, respectively. CIA+TG is the collagen-induced arthritis group treated with 7.89 mg/kg tripterygium glycosides.
